# Special Issue “Biochemical and Molecular Approaches to Postharvest Research”: An Overview

**DOI:** 10.3390/ijms252111807

**Published:** 2024-11-03

**Authors:** Isabel Lara, Josep M. Puche Fontanilles

**Affiliations:** 1 Departament de Química, Física i Ciències Ambientals i del Sòl, Universitat de Lleida (UdL), 25198 Lleida, Spain; 2 AGROTÈCNIO Center, 25198 Lleida, Spain; 3 Institut Català d’Arqueologia Clàssica, (ICAC), 43003 Tarragona, Spain; jpuche@icac.cat; 4 Departament de Ciències de l’Antiguitat i de l’Edat Mitjana, Universitat Autónoma de Barcelona (UAB), 08193 Bellaterra, Spain

“*O fortunatos nimium, sua si bona norint agricolas!*”

Virgil (Georgics II, 458)

## 1. Introduction

Postharvest changes in fruit and vegetable quality involve numerous, complex, and often interlinked metabolic pathways and physiological events. Because metabolic activity remains high after harvest, the control of these events is challenging. For this reason, fruits and vegetables are highly perishable commodities, and the deterioration of their quality is the cause of important economic losses, particularly when shipped to faraway destination markets.

The main goal of postharvest research is to delay senescence and spoilage during shelf life and storage, while preserving and even favoring desirable quality traits. However, fruit and vegetables are also remarkably diverse in their structure, physiology, and tolerance to environmental conditions. In addition, the use of agrochemicals is becoming ever more restricted by local and international regulations and unacceptable to the final consumers of the produce. Therefore, postharvest handling and storage procedures need to be tailored to each specific commodity.

To achieve these goals, a deeper and more comprehensive knowledge of the physiological, biochemical, and molecular basis of fruit and vegetable quality is required, and the application of advanced analysis techniques will be paramount to aid the in-depth understanding of postharvest changes. The Special Issue “Biochemical and Molecular Approaches to Postharvest Research” was launched with the aim of offering an overview of recent research work in this area.

Twelve manuscripts were submitted for publication in this Special Issue, five of which were accepted after peer-review. The articles finally compiled in this Special Issue delve into the complex biological and physiological processes that influence the quality of plant produce over postharvest life. These studies considered climacteric (pear and saskatoon berry) and non-climacteric (orange and table grape) fruit species, as well as cassava, as a representative example of an important tuberous-root crop. Reported research examined the biochemical and molecular mechanisms underlying produce deterioration after harvest, and explored the potential of different postharvest treatments (abscisic acid, ozone, and CO_2_) to delay quality loss and hence to extend the shelf life of produce. The aim of this Editorial is to summarize and provide a joint discussion of these contributions, which are listed below:

- He et al. Changes in α-farnesene and phenolic metabolism and the expression of associated genes during the development of superficial scald in two distinct pear cultivars. https://doi.org/10.3390/ijms232012088
- Lafuente and González-Candelas. The role of ABA in the interaction between citrus fruit and *Penicillium digitatum*. https://doi.org/10.3390/ijms232415796
- Li et al. Comparative transcriptome profiling of cassava tuberous roots in response to postharvest physiological deterioration. https://doi.org/10.3390/ijms24010246
- Matłok et al. Effects of post-harvest ozone treatment on some molecular stability markers of *Amelanchier alnifolia* Nutt. fruit during cold storage. https://doi.org/10.3390/ijms231911152
- Romero et al. Short-term gaseous treatments improve rachis browning in red and white table grapes stored at low temperature: Molecular mechanisms underlying its beneficial effect. https://doi.org/10.3390/ijms232113304

## 2. Understanding Produce Perishability and Quality Deterioration After Harvest

Superficial scald is a common physiological disorder affecting the quality of apple (*Malus* × *domestica* Borkh.), as well as both European (*Pyrus communis* L.) and Asian (*P. serotina* Rehd.; *P.* × *bretschneideri* Rehd.) pear fruit during long-term cold storage. Some cultivars, such as ‘Granny Smith’ apple or ‘Anjou’ pear, are particularly prone to develop this alteration. The disorder manifests on fruit skin as brown patches that develop upon removal from cold storage to ambient temperature [1]; this is the cause of important economic losses owing to detrimental effects on fruit appearance despite the fact that the fruit flesh remains of good eating quality.

In this context, He et al. explored the development of superficial scald in two pear varieties (‘Wujiuxiang’ and ‘Yali’)—a European and an Asian pear cultivar, respectively, characterized by different susceptibilities to the disorder. Whereas the incidence of superficial scald in fruit of the ‘Yali’ pear (*P.* × *bretschneideri* Rehd.) is low, ‘Wujiuxiang’ pear (*P. communis* L.), a hybrid of ‘Yali’ and ‘Bartlett’ (*P. communis* L.) cultivars, suffers from high susceptibility to the disorder. The expression patterns of several genes associated with the biosynthesis of key volatile compounds such as α-farnesene and its oxidation products, conjugated trienols (CTols), with the phenolic metabolism pathway and with the permeability of cell membranes, were assessed in both cultivars. The results showed that the expression of the genes involved in α-farnesene and CTol metabolism and in the biosynthesis of phenolics was substantially up-regulated in fruit of the scald-sensitive ‘Wujiuxiang’ cultivar upon the onset of superficial scald development. The development of the disorder was also accompanied by higher values for cell membrane permeability, higher contents of catechin and epicatechin, and higher expression levels of related genes. In contrast, lower expression levels of phenolic-synthesis-related and polyphenol oxidase genes in parallel to lower contents of chlorogenic acid, arbutin, and isorhamnetin-3-3-glucoside were also observed in fruit of the susceptible cultivar. The results indicated that the onset and progression of superficial scald were associated with oxidation processes, manifested in the accumulation of CTols, cell membrane breakdown, and the oxidation of phenolics.

The storability and shelf life of fruit produce can also be severely hampered by fungal infections. A major source of postharvest decay is green mold (*Penicillium digitatum* (Pers.) Sacc.), which is a fungus species that displays high host specificity, as it infects mainly citrus fruit species [2]. The postharvest control of the disease by fungicide application has led to the emergence of resistant *P. digitatum* populations, in addition to posing challenges to the fruit industry related to the toxicity and environmental risks of such treatments. Accordingly, there is great interest in understanding the mechanisms involved in induced resistance to fungal infection with the purpose of developing new alternatives for the control of *P. digitatum* infection. Therefore, Lafuente and González-Candelas investigated a potential role of abscisic acid (ABA) in enhancing the resistance of citrus fruit to infection by *P. digitatum*. ABA is involved in a plethora of developmental, environmental, and hormonal cross-talk responses [3]. In this work, ABA was demonstrated to be a key factor for resistance to fungal infection during the postharvest period, by showing that higher ABA levels were associated with more effective defense mechanisms in infection-resistant citrus varieties.

In addition to fruit commodities, tuberous roots are also economically important crops worldwide. Cassava (*Manihot esculenta* Crantz), for example, serves as a basic food source to many millions of people worldwide, particularly in tropical and subtropical areas. Beyond its value as a staple food, cassava also reportedly displays remarkable health-promoting properties, including anti-tumoral, anti-inflammatory, hypocholesterolemic, and anti-bacterial activities, among others [4]. However, cassava tubers are subject to rapid physiological postharvest deterioration (PPD), which hinders their handling potential and is the cause of huge economic losses. Therefore, the extension of the storage potential of cassava is the object of intense research focus. In this regard, Li et al. investigated transcriptional regulation in cassava during postharvest storage in order to understand the key molecular mechanisms related to PPD. Two cassava cultivars displaying differential resistance to PPD (‘SC8’ and ‘RYG1’, a sensitive and a tolerant variety, respectively) were kept at around 29 °C for up to 6 weeks after harvest. Delayed PPD in ‘RYG1’ tubers was related to lower levels of reactive oxygen species (ROS), associated with better preservation of the cell wall structure and higher contents of soluble sugars. Transcriptome profile analyses suggested that complex mechanisms, such as photosynthesis regulation, the processing of endoplasmic reticulum proteins, phenolics metabolism, and phytohormone-signaling pathways, are also involved in PPD tolerance.

## 3. The Interplay Between Biochemical Pathways and External Treatments

For the development of novel postharvest strategies, sustainability must be a critical consideration. There is a need for global agricultural practices to reduce their reliance on agrochemicals and adapt to stricter legal regulations. These concerns have led to the search for innovative solutions to postharvest challenges. Some of the articles compiled in this Special Issue explored hormonal and gaseous treatments as potentially effective, yet environmentally friendly, alternative strategies to extend the shelf life of fruit produce.

The work by Lafuente and González-Candelas not only demonstrated a key role for ABA in the resistance to postharvest fungal infection, but it also showed that the application of exogenous ABA can actually enhance the resistance of citrus fruit to infection by *P. digitatum* in susceptible cultivars. To prove this, the authors considered two orange (*Citrus sinensis* (L.) Osbeck) cultivars, namely ‘Navelate’ and its ABA-deficient mutant ‘Pinalate’, which is less resistant to fungal infection. ABA was applied to ‘Pinalate’ fruit by dipping them for 2 min in a 1 mM ABA solution. Interestingly, exogenous ABA restored resistance capacity in ‘Pinalate’ oranges to similar levels to those in ‘Navelate’. Remarkably, applied ABA up-regulated, among others, a pathogenesis-related thaumatin protein and a bifunctional inhibitor/lipid-transfer protein (LTP), which are relevant in plant immunity. Moreover, the expression of several genes, including some encoding oxidative stress 3 (*OXS3*) proteins and chlorophyllase 1 (*CLH1*), were down-regulated in ABA-treated ‘Pinalate’ fruit. This is noteworthy, since the absence of OXS3 activates ABA-responsive genes in plants. These data illustrate that the manipulation of hormonal levels may boost plant natural defenses, resulting in a reduced postharvest decay incidence.

Two of the studies investigated the potential benefits of gaseous treatments for the extension of the commercial life of fruit. Matłok et al. evaluated the potential of ozone treatments on selected chemical and mechanical parameters of saskatoon berries (*Amelanchier alnifolia* Nutt.) kept in storage. Saskatoon berries are an under-utilized climacteric fruit species within the *Rosaceae* family that shows outstanding nutritional and health-promoting traits, since these fruits are an excellent source of vitamins, minerals, sugars, and antioxidant compounds [5]. The shelf life potential of saskatoon berries is short, and their properties deteriorate rapidly after harvest. The authors of this study were interested in assessing the effects of ozonation on mechanical properties, microbial load, and selected chemical properties in two saskatoon berry cultivars, with particular focus on antioxidant compounds. Ozone treatment (10 ppm) was applied for 15 or 30 min to the fruit of ‘Smoky’ and ‘Martin’ cultivars, which were subsequently kept at 8 ◦C for 7 days. Treated fruit preserved higher contents of bioactive compounds, had lower water loss rates, and lower microbial load on the fruit surface, leading to significantly decreased spoilage during the experimental storage period.

On the other hand, carbon dioxide (CO_2_) shocks and storage under CO_2_-enriched atmospheres have been observed to improve the storability of some fruit commodities [6]. Sensitivity to high CO_2_ levels is highly variable across species and cultivars, and high CO_2_ concentrations may induce fermentative processes, unwanted flavors, and physiological disorders in sensitive fruit cultivars. However, such conditions may also have desirable effects on fruit quality in some commodities, including lowered respiration rates with a concomitant reduction in the oxidative damage of fruit tissues, chlorophyll degradation, and ethylene sensitivity, which, in turn, results in delayed ripening and senescence. Accordingly, Romero et al. explored the suitability of a short-term CO_2_ treatment to reduce rachis browning, a key factor in the loss of the visual and functional quality of grapes during cold storage. In a similar manner to the superficial scalding of apples and pears, rachis browning affects freshness perception and acceptance by potential purchasers. In this work, clusters of a red and a white cultivar (‘Red Globe’ and ‘Superior Seedless’, respectively) of table grapes (*Vitis vinifera* L.) were exposed to a 20 kPa CO_2_ shock and then stored at 0 °C for 28 days under normal air. CO_2_-treated berries displayed significantly lower oxidative stress levels and contents of phenolics in the rachis in comparison with controls. The treatment activated responses related to non-enzymatic and enzymatic antioxidant systems. These effects included the up-regulated expression of genes related to the enzymatic antioxidant system and a lower content of phenolics. The oxidative processes in the rachis of non-treated berry clusters were thus prevented, as well as the induction of genes encoding for proteins related to cell wall disassembly.

## 4. Conclusions

The studies compiled in this Special Issue provide valuable insights into the complexities of postharvest biology, and hint at potential practical strategies for extending the shelf life period of fruit and tuber commodities, thus minimizing food losses. The experimental methodologies included molecular, biochemical, and metabolomic approaches, and the issues of perishability and disease resistance were addressed in a handful of relevant crops. Taken together, the results indicate the importance of the oxidative status for enhanced storage potential and resistance to infections. This may provide clues to target metabolic pathways whose modulation may delay postharvest spoilage.
